# CXC Chemokine/Receptor Axis Profile and Metastasis in Prostate Cancer

**DOI:** 10.3389/fmolb.2020.579874

**Published:** 2020-10-15

**Authors:** Naoya Nagaya, Geun Taek Lee, Shigeo Horie, Isaac Yi Kim

**Affiliations:** ^1^Section of Urologic Oncology, Rutgers Robert Wood Johnson Medical School, Rutgers Cancer Institute of New Jersey, New Brunswick, NJ, United States; ^2^Department of Urology, Juntendo University Graduate School of Medicine, Tokyo, Japan

**Keywords:** chemokine, chemokine receptor, CXCR3, CXCL10, prostate cancer, metastasis, lymph node, bone

## Abstract

In this study, the effects of the CXC chemokine/receptor axis on lymph node and distant metastases of prostate cancer (PC) were analyzed. Further, mRNA expression data of metastatic PC were extracted from the Stand Up To Cancer–Prostate Cancer Foundation Dream Team database and differences between metastatic sites were comprehensively analyzed. CXC chemokine/receptor mRNA expression data of primary PC included in the Cancer Genome Atlas were used to analyze the relationships of CXC chemokine/receptor expression with lymph node metastasis and cancer progression. In metastatic PC, significantly higher expression of ELR^+^ CXC chemokines/receptors and significantly lower expression of ELR^−^ CXC chemokines/receptors were observed in bone metastases relative to lymph node metastases. In primary PC, significantly higher ELR^−^ CXC chemokine/receptor expression and significantly lower ELR^+^ CXC chemokine/receptor expression were observed in patients with lymph node metastasis relative to those without. Multivariate logistic regression analysis identified CXCL10 expression as an independent predictor of lymph node metastasis. Furthermore, the log-rank test results revealed that co-expression of CXCL10/CXCR3 was associated with postoperative recurrence. These findings demonstrate heterogeneous expression of CXC chemokine/receptor genes in primary PC as well as differences in expression patterns according to the metastatic site.

## Introduction

In most cases, prostate cancer (PC) is localized and can be cured by surgical resection. In some men, however, PC metastasizes to the lymph nodes or to distant sites, primarily the bone (Muralidhar et al., 2015). Because the survival of a cancer relies on the ability to metastasize, an understanding of the mechanism underlying PC metastasis could potentially yield new diagnostic biomarkers and treatment strategies.

Leukocyte transport is crucially regulated by chemokines and their receptors, and tumor cell migration and metastasis are controlled through a similar chemokine-dependent process (Muller et al., 2001). Particularly, the CXC chemokine subgroup participates in the regulation of tumor-associated angiogenesis and cancer cell metastasis (Keeley et al., 2010; Sarvaiya et al., 2013). CXC chemokines are subcategorized based on the presence or absence of the glutamate-leucine-arginine (ELR) motif within the three amino acid residues proximal to the CXC sequence (Table 1). ELR^+^ CXC chemokines are associated with neutrophil chemotaxis and ELR^−^ CXC chemokines with lymphocyte chemotaxis.

**Table 1:** List of the CXC Chemokine ligands and chemokine receptors.

**E-L-R amino acid motif**	**Chemokine**	**Receptor**
ELR^−^	CXCL4 (PF4)	CXCR3B
	CXCL9	CXCR3A, CXCR3B
	CXCL10	CXCR3A, CXCR3B
	CXCL11	CXCR3A, CXCR3B, CXCR7 (ACKR3)
	CXCL12	CXCR4, CXCR7 (ACKR3)
	CXCL13	CXCR 5>> CXCR3
	CXCL16	CXCR6
ELR^+^	CXCL1	CXCR2
	CXCL2	CXCR2
	CXCL3	CXCR2
	CXCL5	CXCR2 >> CXCR1
	CXCL6	CXCR2 > CXCR1
	CXCL7 (PPBP)	CXCR1, CXCR2
	CXCL8 (IL-8)	CXCR1, CXCR2
	CXCL14	Unknown
	CXCL15	Unknown

*CXC chemokines and corresponding receptors are categorized by the presence or absence of the glutamate-leucine-arginine (ELR) motif within the three amino acid residues proximal to the CXC sequence*.

In the tumor environment, the CXC chemokine/receptor axis induces tumor cell migration in an organ-specific manner through an inherent cell migration system, and CXC chemokines are involved in the creation of a microenvironment that supports the growth of metastatic tumor cells (Kang et al., 2003; Kawada et al., 2004; Zlotnik et al., 2011; Sarvaiya et al., 2013; Chow and Luster, 2014). These findings suggest that tumor metastasis to specific organs is supported by the interactions of chemokine receptors on cancer cells with ligands in target organs. Although several studies have shown that the CXC chemokine/receptor axis promotes tumor metastasis and growth in PC (Murphy et al., 2005; Shamaladevi et al., 2009; Lillard et al., 2010; Dubrovska et al., 2012; Salazar et al., 2013; Shen and Cao, 2015), the role of the CXC chemokine/receptor axis in the organ-specific metastasis of PC remains unclear.

Given the heterogeneous nature of PC (Tolkach and Kristiansen, 2018), we hypothesized that CXC chemokine/receptor expression in patients with PC might also be heterogeneous and would depend on the site of metastasis, and metastasis sites also display abundant CXC chemokines for corresponding CXC chemokine receptors. Therefore, the aim of the present study was to comprehensively examine the expression patterns of CXC chemokines/receptors using a dataset of patients with metastatic PC retrieved from the Stand Up To Cancer–Prostate Cancer Foundation (SU2C–PCF) Dream Team database (Abida et al., 2019) and to identify differences in expression patterns among the metastatic sites. Further, the expression patterns of CXC chemokines/receptors in primary PC lesions from the Cancer Genome Atlas (TCGA) dataset (Hoadley et al., 2018) were examined as well as the relationships of CXC chemokine/receptor expression patterns with lymph node metastasis and cancer progression.

## Methods

### Data Source

The RNA-seq data and clinical information of patients with metastatic castration-resistant PC included in the SU2C–PCF Dream Team database as well as the RNA-seq data and clinical information of patients with primary PC included in the TCGA database were downloaded from cBioPortal for Cancer Genomics (https://www.cbioportal.org/). A flowchart of the data retrieval process is shown in Supplementary Figure 1.

### Gene Set Enrichment Analysis (GSEA)

GSEA (Subramanian et al., 2005) was used to identify differences in the enrichment of the Kyoto Encyclopedia of Genes and Genomes (KEGG) pathways (Kanehisa and Goto, 2000) between different metastatic sites with the use of data retrieved from the SU2C–PCF Dream Team database.

### mRNA Expression Analysis

mRNA expression levels in metastatic castration-resistant PC were compared by metastasis site. mRNA expression levels in primary PC were compared between patients with and without lymph node metastases. The Gene Expression Profiling Interactive Analysis 2 Web tool (http://gepia2.cancer-pku.cn/#index) (Tang et al., 2019) was used to identify the correlations between the gene expression levels of CXC chemokines and the corresponding receptors. Spearman correlation analysis was used to determine the probability (*p*) values.

### Lymph Node Metastasis-Predictive Model in the TCGA Cohort

Logistic regression analysis was used to analyze the odds ratios for the presence or absence of lymph node metastases. The preoperative prostate-specific antigen (PSA) concentration, clinical tumor stage, and Gleason scores were used to determine the PC risk preoperatively and were used together with the CXC chemokine and receptor expression data as explanatory variables in a comparison of models to predict the existence of lymph node metastases. In the TCGA dataset, Gleason scores were not available for needle core biopsies, but rather only for radical prostatectomy specimens. However, upgraded Gleason scores of radical prostatectomy specimens compared with needle core biopsies have been reported (Nayyar et al., 2010). To alleviate the effect of possible upgrading, only the primary (most predominant) Gleason score was used as a variable in the predictive model. The variance inflation factor was used as an index to detect multiple collinearities among variables. Receiver operating characteristic curve analysis was used to determine the diagnostic value of the predictive model.

### Kaplan–Meier Analysis of the Survival of Patients With Primary PC

The associations between CXC chemokines/receptors and disease-free survival in the TCGA cohort were analyzed. Disease relapse was either biochemical recurrence or radiological tumor recurrence/metastasis. The *p*-values for survival were calculated using the log-rank test. The log-rank test for trends was used to identify the existence of a linear trend between column order and median survival.

### Statistical Analysis

All statistical analyses were performed with EZR (Saitama Medical Center, Jichi Medical University, Saitama, Japan), which is a graphical user interface for R (version 3.5.2.) (Kanda, 2013). The significance of statistical differences of non-normally distributed continuous variables between the groups was evaluated using Mann–Whitney's *U*-test. All statistical tests were two-sided and *p*-values of <0.05 were considered statistically significant.

## Results

### Difference in KEGG Pathway Enrichment According to PC Metastasis Sites

**Figure 1:**
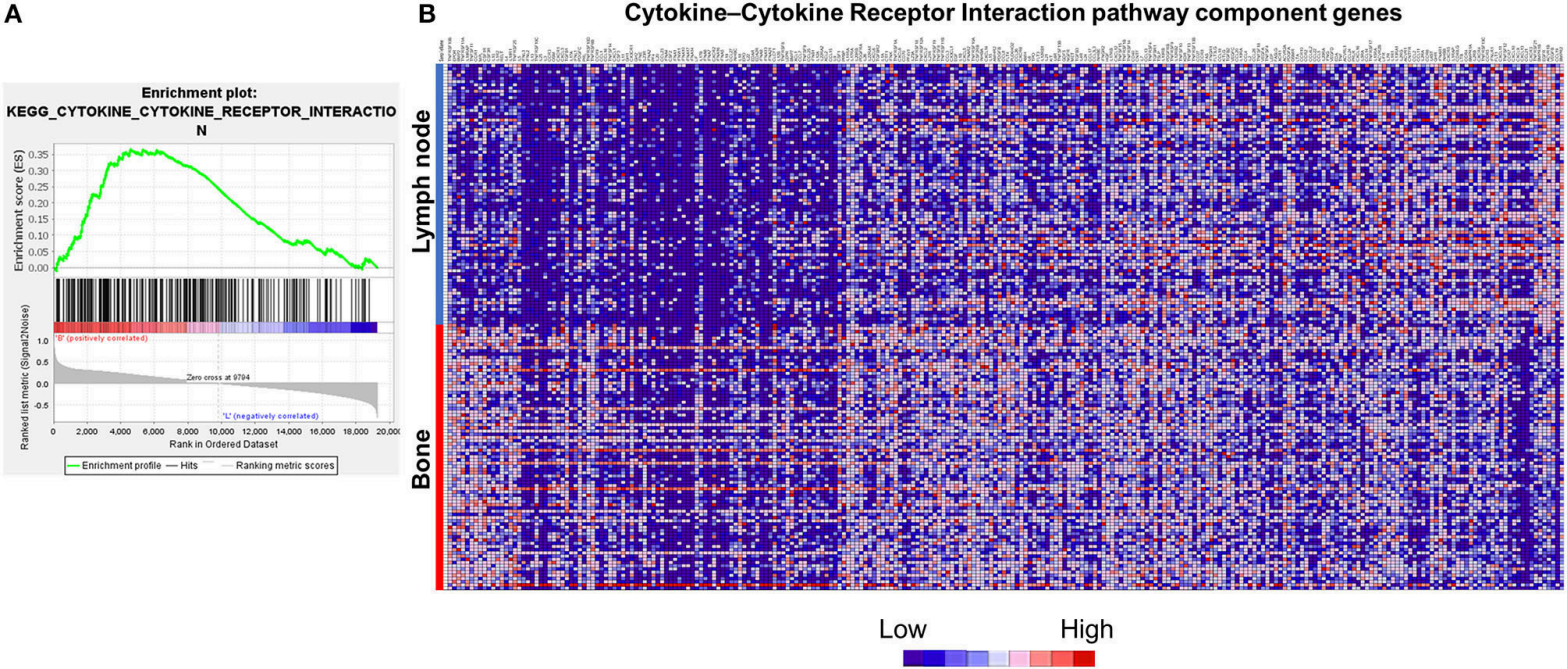
Differences in KEGG cytokine–cytokine receptor interaction pathway enrichment at different PC metastatic sites. **(A)** Gene set enrichment analysis (GSEA) of the cytokine–cytokine receptor interaction pathway components in bone and lymph node metastases. Distribution of 262 cytokine–cytokine receptor interaction pathway genes. **(B)** Heatmap of genes related to the cytokine–cytokine receptor interaction pathway. The heatmap was generated from RNA expression data (*Z*-score) by GSEA. GSEA-derived Heatmap represents the expression levels as colors. The range of colors (red, pink, light blue, dark blue) shows the range of expression values (high, moderate, low, lowest). The horizontal axis indicates ranked genes and the vertical axis shows samples according to the metastasis site (lymph node or bone). Cytokine–Cytokine Receptor Interaction pathway component genes in the figure are consistent with Supplemental Table 2, from left to right. IL3RA, CRLF2, CSF2RA, and IL9R are not included in the figure because mRNA expression data were not available.

mRNA expression profiles were comprehensively examined using a dataset of patients with metastatic PC retrieved from the SU2C–PCF Dream Team database. Tumors included in the SU2C–PCF Dream Team dataset were collected from various sites including the bone, lymph node, liver, prostate, adrenal gland, and other soft tissues (Supplementary Figure 2). Of these, the bone and lymph nodes were identified as the main metastatic sites of PC. The SU2C–PCF Dream Team database contained approximately the same number of samples at both sites. There were no significant differences in the PSA concentrations and Gleason scores between metastases specimens of the bone and lymph nodes (Supplementary Figure 2). Therefore, GSEA was performed to identify differences in KEGG pathway enrichment between metastases sites of the bone (*n* = 83) and lymph nodes (*n* = 81). The analysis revealed differences in several pathways, including the olfactory transduction pathway, neuroactive ligand receptor interaction pathway, Extracellular matrix (ECM) receptor interaction pathway, and cytokine–cytokine receptor interaction pathway (Supplementary Table 1). The expression levels of genes in leading-edge subsets of the cytokine–cytokine receptor interaction pathway, including CXCL1, CXCL3, CXCL6, CXCR1, and CXCR2, were higher in bone metastasis than in lymph node metastasis (Figures 1A,B, Supplementary Table 2).

### Differences in CXC Chemokine/Receptor Gene Expression Profiles by PC Metastasis Sites

**Figure 2:**
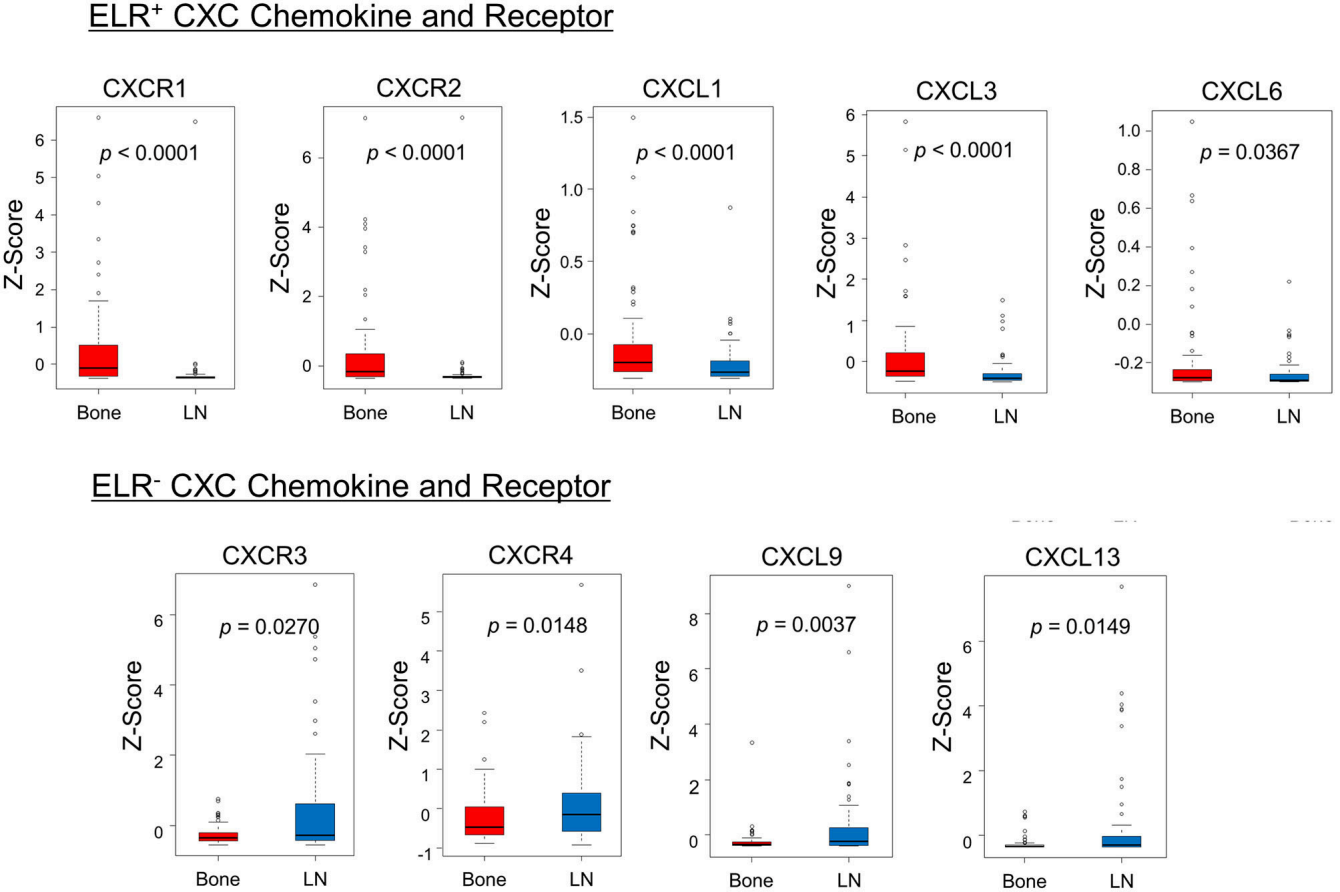
Comparison of the expression patterns of CXC chemokines/receptors. The box plots indicate mRNA expression levels of individual samples. Significantly higher expression levels of ELR^+^ CXC chemokine/receptor genes (CXCL1, CXCL3, CXCL6, CXCR1, and CXCR2) and significantly lower expression levels of ELR^−^ CXC chemokine/receptor genes (CXCL9, CXCL13, CXCR3, and CXCR4) were observed in bone metastases relative to lymph node metastases. mRNA expression profiles of bone and lymph node metastases are indicated in red and blue, respectively. Statistically significant differences in mRNA expression levels between the groups were identified using Mann–Whitney's *U*-test after confirmation of non-normal distributions.

Furthermore, the mRNA expression profiles of CXC chemokine/receptor genes in the cytokine–cytokine receptor interaction pathway gene set, leading-edge genes, and other CXC chemokine/receptor genes were compared. Comparative analysis of the CXC chemokine/receptor genes revealed significantly higher expression levels of genes encoding ELR^+^ CXC chemokines/receptors (CXCL1, CXCL3, CXCL6, CXCR1, and CXCR2) in bone metastases relative to lymph node metastases. In contrast, the expression levels of genes encoding ELR^−^ CXC chemokines/receptors (CXCL9, CXCL13, CXCR3, and CXCR4) were significantly higher in lymph node metastases (Figure 2). The CXC chemokine/receptor gene expression levels at all metastatic sites are presented in Supplementary Figure 3.

### Differences in CXC Chemokine/Receptor Gene Expression Levels Between Primary Tumors and Lymph Nodes

**Figure 3:**
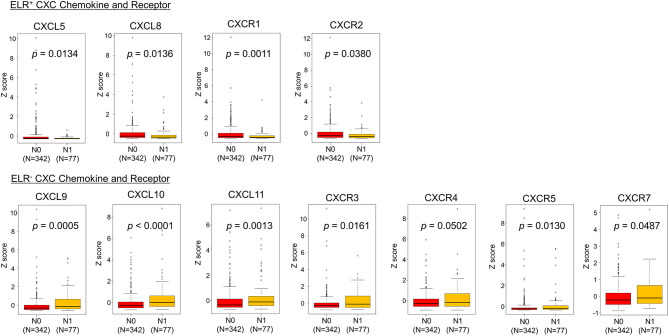
Comparisons of CXC chemokine/receptor gene expression levels in primary PC. The box plots indicate the mRNA expression profiles of individual samples. Significantly higher expression of ELR^−^ CXC chemokine/receptor genes (CXCL9, CXCL10, CXCL11, CXCR3, CXCR5, and CXCR7) and significantly lower expression of ELR^+^ CXC chemokine/receptor genes (CXCL5, CXCL8, CXCR1, and CXCR2) were observed in patients with lymph node metastasis-positive (N1) PC than in those with lymph node metastasis-negative (N0) PC. CXCR4 (ELR^−^ CXC chemokine receptor) tended to be higher (*p* = 0.0502) in N1 patients than in N0 patients. Statistically significant differences in mRNA expression levels between the groups were identified using Mann–Whitney's *U*-test after confirmation of non-normal distributions.

Next, the TCGA dataset was used to verify the potential relationships between CXC chemokine/receptor gene expression patterns in primary PC and the existence of lymph node metastasis. In the TCGA, positive lymph node metastasis was diagnosed histologically. The CXC chemokine/receptor gene expression levels were compared between lymph node metastasis-negative (N0; *n* = 342) and lymph node metastasis-positive (N1; *n* = 77) patients with verifiable gene expression data. In this analysis, significantly higher ELR^−^ CXC chemokine/receptor expression (CXCL9, CXCL10, CXCL11, CXCR3, CXCR5, and CXCR7) and significantly lower ELR^+^ CXC chemokine/receptor expression (CXCL5, CXCL8, CXCR1, and CXCR2) were observed in N1 patients than in N0 patients. CXCR4 (ELR^−^ CXC chemokine receptor) expression tended to be higher (*p* = 0.0502) in N1 patients than in N0 patients (Table 2 and Figure 3).

**Table 2:** CXC chemokine and receptor expression in primary PC.

**CXC chemokine/receptor**		**N0 (*n* = 342)**	**N1 (*n* = 77)**	**N0 vs. N1**
		**Transcript per million (Lower quartile—upper quartile)**		**(*p*-value)**
ELR^−^ Chemokine	CXCL4	−0.46 (−0.46 to 0.10)	−0.46 (−0.46 to 0.31)	0.0639
	CXCL9	−0.29 (−0.45 to −0.01)	−0.12 (−0.39 to −0.65)	0.0005
	CXCL10	−0.30 (−0.50 to 0.03)	0 (−0.38 to 0.62)	<0.0001
	CXCL11	−0.36 (−0.54 to 0.11)	−0.13 (−0.42 to 0.32)	0.0013
	CXCL12	−0.19 (−0.56 to 0.21)	−0.25 (−0.67 to 0.12)	0.1330
	CXCL13	−0.24 (−0.27 to −0.13)	−0.23 (−0.26 to −0.07)	0.2210
	CXCL16	−0.06 (−0.67 to 0.50)	0.11 (−0.72 to 0.64)	0.8300
ELR^−^ Chemokine	CXCR3	−0.26 (−0.49 to 0.04)	−0.09 (−0.49 to 0.87)	0.0161
Receptor	CXCR4	−0.26 (−0.55 to0.16)	−0.18 (−0.53 to 0.67)	0.0502
	CXCR5	−0.24 (−0.30 to −0.13)	−0.21 (−0.29 to 0.06)	0.0130
	CXCR6	−0.20 (−0.48 to 0.22)	−0.06 (−0.51 to 0.40)	0.3760
	CXCR7	−0.22 (−0.48 to 0.18)	−0.11 (−0.43 to 0.65)	0.0487
ELR^+^ Chemokine	CXCL1	−0.29 (−0.42 to 0)	−0.32 (−0.43 to −0.11)	0.4960
	CXCL2	−0.25 (−0.31 to −0.03)	−0.28 (−0.34 to −0.15)	0.1090
	CXCL3	−0.21 (−0.26 to −0.03)	−0.23 (−0.26 to −0.12)	0.2320
	CXCL5	−0.25 (−0.29 to −0.11)	−0.28 (−0.30 to −0.21)	0.0134
	CXCL6	−0.26 (−0.43 to 0.11)	−0.30 (−0.49 to 0.02)	0.1170
	CXCL7	−0.11 (−0.11 to −0.04)	−0.11 (−0.11 to −0.02)	0.1790
	CXCL8	−0.26 (−0.40 to 0.08)	−0.34 (−0.45 to −0.15)	0.0136
ELR^+^ Chemokine	CXCR1	−0.29 (−0.47 to 0.09)	−0.42 (−0.50 to −0.23)	0.0011
Receptor	CXCR2	−0.28 (−0.50 to 0.17)	−0.39 (−0.55 to −0.09)	0.0380

*The CXC chemokine/receptor gene expression levels were compared between lymph node metastasis-negative (N0; n = 342) and lymph node metastasis-positive (N1; n = 77) patients. Statistically significant differences in mRNA expression between the groups were identified using Mann–Whitney's U-test after confirmation of non-normal distributions*.

### Correlations of CXC Chemokine and Receptor Expression Levels in PC

**Figure 4:**
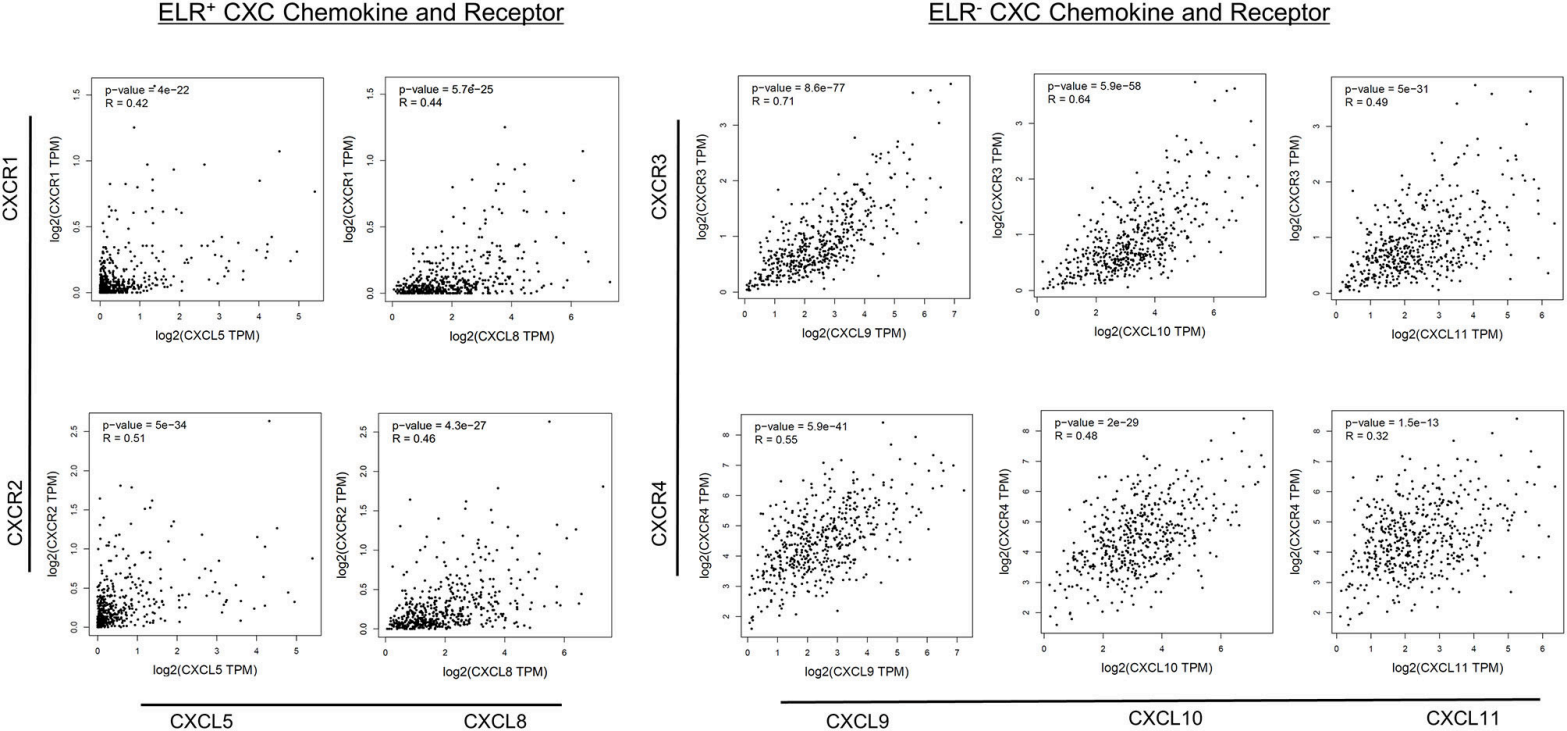
Correlations between CXC chemokine/receptor expression levels in primary PC. Significant positive correlations in the expression levels of ELR^+^ CXC (CXCL5, CXCL8, CXCR1, CXCR2) and ELR^−^ CXC (CXCL9, CXCL10, CXCL11, CXCR3, and CXCR4) chemokines/receptors were observed (*p* < 0.01).

Next, correlations among ELR^−^ CXC and ELR^+^ CXC chemokines/receptors were examined. The expression levels of all ELR^+^ CXC and ELR^−^ CXC chemokines, with the exception of CXCR7, were significantly and positively correlated with the expression levels of the respective receptors (R = 0.32–0.71), whereas moderate correlations were observed between CXCR3 and CXCL9 as well as between CXCR3 and CXCL10 (R > 0.60; Figure 4).

### Associations of CXC Chemokine/Receptor Expression Patterns in Primary PC and the Presence of Lymph Node Metastasis

First, the significance of the preoperative PSA concentration, clinical tumor stage, and primary Gleason score was analyzed, as these factors are commonly used to assess disease progression risk (low, intermediate, or high risk) during preoperative evaluation, along with the expression patterns of ELR^−^ CXC and ELR^+^ CXC chemokines/receptors by univariate logistic regression analysis to predict the presence of lymph node metastasis. Notably, the preoperative PSA concentration, clinical tumor stage, and primary Gleason score were identified as significant predictors of lymph node metastasis, as were all evaluated CXC chemokine/receptors, with the exceptions of CXCR2 and CXCL8 (Table 3A).

**Table 3A:** Unadjusted odds ratio in lymph node metastasis predictive model.

	**Variable**	**Odds ratio**	***p*-value**	**AUC**
	PSA (Preoperative)	1.06 (1.02–1.10)	0.0070	0.79 (0.67–0.91)
	Primary Gleason Score	3.07 (2.27–4.17)	<0.0001	0.76 (0.73–0.81)
	Clinical Tumor stage	1.23 (1.04–1.45)	0.0158	0.59 (0.51–0.66)
ELR^+^ Chemokine Receptor	CXCR1	0.51 (0.31–0.86)	0.0115	0.61 (0.55–0.68)
	CXCR2	0.68 (0.45–1.02)	0.0595	0.57 (0.50–0.64)
ELR^+^ Chemokine	CXCL5	0.16 (0.03–0.81)	0.0276	0.59 (0.52–0.65)
	CXCL8	0.83 (0.59–1.18)	0.3110	0.59 (0.51–0.66)
ELR^−^ Chemokine Receptor	CXCR3	1.34 (1.08–1.65)	0.0066	0.58 (0.50–0.66)
	CXCR4	1.33 (1.08–1.63)	0.0075	0.57 (0.49–0.64)
	CXCR5	1.24 (1.01–1.54)	0.0417	0.59 (0.51–0.66)
	CXCR7	1.40 (1.07–1.83)	0.0127	0.57 (0.49–0.64)
ELR^−^ Chemokine	CXCL9	1.32 (1.08–1.61)	0.0077	0.62 (0.55–0.69)
	CXCL10	1.50 (1.22–1.85)	0.0001	0.64 (0.57–0.71)
	CXCL11	1.34 (1.10–1.63)	0.0039	0.61 (0.55–0.68)

*AUC, Area under the curve; PSA, Prostate-Specific Antigen*.

*The significance of the preoperative PSA concentration, clinical tumor stage, and primary Gleason score with the expression of ELR^−^ CXC and ELR^+^ CXC chemokines/receptors was analyzed by univariate logistic regression analysis*.

Second, multivariate logistic regression analysis was performed to evaluate independent predictors of lymph node metastasis in PC with the use of clinical information (preoperative PSA and primary Gleason score) as explanatory variables (Model 1). The clinical stage was excluded as an explanatory variable because of the low AUC value of this parameter. One ELR^+^ CXC chemokine, one ELR^−^ CXC chemokine, and the corresponding receptors among those with the highest area under the curve (AUC) values by univariate analysis (CXCL5/CXCR1 and CXCL10/CXCR3; Model 2) were also included to determine if CXC chemokine expression affects the prediction of lymph node metastasis. The results of Model 1, which were based solely on clinical information (preoperative PSA and primary Gleason score), clarified that these clinical variables were independent predictors of the existence of lymph node metastasis (Table 3B). Moreover, the results of Model 2 further identified CXCL10 as an independent predictor of the existence of lymph node metastases (Table 3C). Model 2 yielded a slightly higher AUC value than Model 1 (0.887 vs. 0.847, respectively).

**Table 3B:** Lymph node metastasis predictive model without CXC chemokines/receptors.

**Variable**	**Odds ratio**	***p-*value**	**VIF**
PSA (Preoperative)	1.05 (1.01–1.09)	0.0241	1.001
Primary Gleason Score	4.52 (1.84–11.1)	0.0009	1.001
Multivariable model 1:			
Lymph Node Stage (N0 or N1) = Primary Gleason Score + PSA (Preoperative)			
*p*-value < 0.001			
AUC = 0.847 (0.765–0.928)			

*AUC, area under the curve; PSA, Prostate-Specific Antigen; VIF, Variance Inflation Factor*.

*Multivariate logistic regression analysis was performed to identify independent predictors of lymph node metastasis in PC using clinical information (preoperative PSA and primary Gleason score) as explanatory variables (Model 1)*.

**Table 3C:** Lymph node metastasis predictive model with CXC chemokines/receptors.

	**Variable**	**Odds ratio**	***p-*value**	**VIF**
	PSA (Preoperative)	1.04 (0.99–1.09)	0.0553	1.070
	Primary Gleason Score	3.86 (1.38–10.8)	0.0100	1.134
ELR^+^ Chemokine Receptor	CXCR1	0.85 (0.33–2.17)	0.7350	1.096
ELR^+^ Chemokine	CXCL5	0.03 (0–3.00)	0.1420	1.141
ELR^−^ Chemokine Receptor	CXCR3	0.67 (0.31–1.43)	0.3010	1.609
ELR^−^ Chemokine	CXCL10	3.47 (1.38–8.75)	0.0083	1.650
Multivariable Model 2:				
Lymph Node Stage (N0 or N1) = Primary Gleason Score + PSA (Preoperative) + CXCR1 + CXCL5 + CXCR3 + CXCL10				
*p*-value < 0.001				
AUC = 0.887 (0.803–0.950)				

*AUC, area under the curve; PSA, Prostate-Specific Antigen; VIF, Variance Inflation Factor*.

*Multivariate logistic regression analysis was performed using clinical information (preoperative PSA and primary Gleason score), one ELR^+^ CXC chemokine, one ELR^−^ CXC chemokine, and the corresponding receptors of those with the highest AUC values by univariate analysis (CXCL5/CXCR1 and CXCL10/CXCR3; Model 2)*.

### Expression Levels of CXCL10 With CXCR3 and PC Prognosis

**Figure 5:**
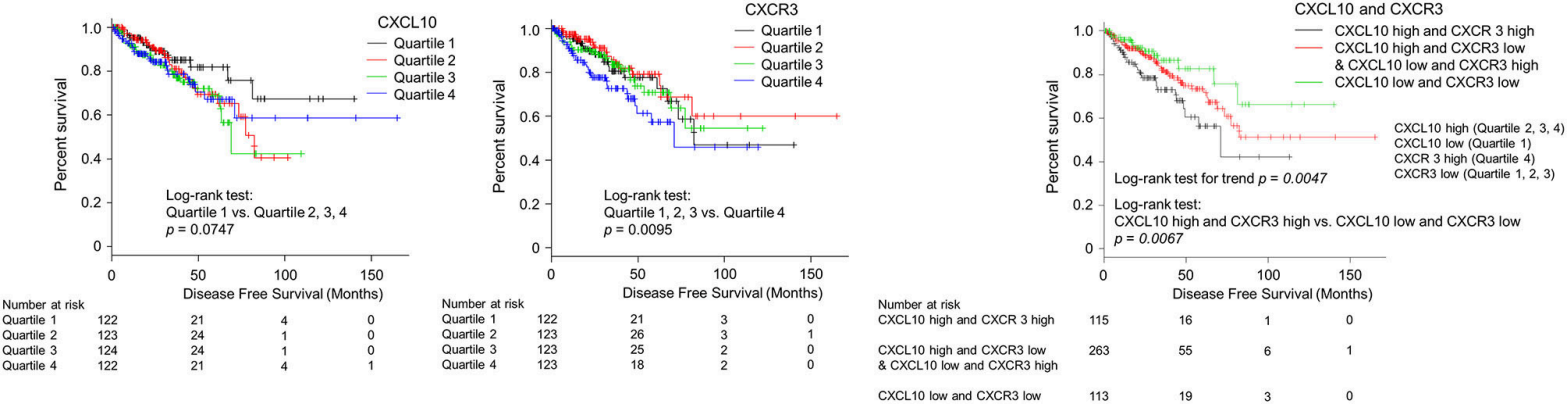
Kaplan–Meier analysis of progression-free survival according to CXCL10 and CXCR3 expression levels in primary PC. Patients with CXCL10 levels between 0–25% were grouped into quartile 1, 25–50% into quartile 2, 50–75% into quartile 3, and 75–100% into quartile 4. CXCR3 levels were also classified into quartiles. Postoperative disease-free survival outcomes for each group are provided in this figure. When CXCL10 was grouped into quartiles, patients in the first quartile had better prognoses than those in other quartiles (*p* = 0.0747). When CXCR3 was grouped into quartiles, patients in the fourth quartile had significantly poorer prognoses than those in the other quartiles (*p* = 0.0095). These results supported designating the first CXCL10 quartile as “low” and the other as “high.” Likewise, the first, second, and third quartiles of CXCR3 were designated as “low” and the fourth quartile as “high.” Patients with both CXCL10 high and CXCR3 high (CXCL10/CXCR3 co-expression) had significantly poorer prognoses than those scored as “low” for both CXCL10 and CXCR3 (*p* = 0.0067).

Survival analysis was performed to assess the value of CXCL10 as an independent predictor of the existence of lymph node metastases with its corresponding receptor CXCR3. Then we analyzed the correlations with disease-free survival outcomes in the TCGA cohort (*N* = 491). To examine the relationship between gene expression of CXC chemokines/receptors and prognosis, disease-free survival in each group was compared by categorizing the values of continuous variables into quartiles. Patients with CXCL10 levels between 0 and 25% were grouped into quartile 1, 25–50% into quartile 2, 50–75% into quartile 3, and 75–100% into quartile 4. CXCR3 levels were also classified into quartiles. Postoperative disease-free survival outcomes for each group are provided in Figure 5. When CXCL10 was grouped into quartiles, patients in the first quartile had better prognoses than those in the other quartiles (*p* = 0.0747). When CXCR3 was grouped into quartiles, patients in the fourth quartile had significantly poorer prognoses than those in the other quartiles (*p* = 0.0095). These results supported designating the first CXCL10 quartile as “low” and the others as “high.” Likewise, the first, second, and third quartiles of CXCR3 were designated as “low” and the fourth quartile as “high.” Patients with both CXCL10 high and CXCR3 high (CXCL10/CXCR3 coexpression) had significantly poorer prognoses than patients with both CXCL10 low and CXCR3 low (*p* = 0.0067). The log-rank test for trends showed that patients with both CXCL10 low and CXCR3 low achieved the best clinical outcomes, whereas those with increased expression levels of either CXCL10 or CXCR3 had medium clinical outcomes, and patients with both CXCL10 high and CXCR3 high had the poorest clinical outcomes (*p* = 0.0047).

## Discussion

The comprehensive analysis of differences in gene expression profiles with respect to metastatic sites in patients with metastatic PC revealed significant differences in CXC chemokine/receptor expression levels between bone and lymph node metastases. Further analysis of CXC chemokine/receptor expression in N0 and N1 patients from the TCGA dataset revealed significant differences in the expression levels of CXC chemokines/receptors in primary tumors between the groups. In addition, the associations of CXC chemokine/receptor expression with the existence of lymph node metastasis were clarified with respect to the survival prognosis of patients with PC.

In this study, the expression levels of ELR^+^ CXC chemokines/receptors (CXCL1, CXCL3, CXCL6, CXCR1, and CXCR2) increased in bone metastases of PC in the cytokine–cytokine receptor interaction pathway gene set. ELR^+^ CXC chemokines have angiogenic properties (Strieter et al., 2005). A previous study of osteosarcoma found that the CXCL6–CXCR1/2 axis contributed to metastasis by inducing epithelial-mesenchymal transition (Liu et al., 2019). Bhawna et al. emphasized the importance of both tumor cell- and host cell-derived CXCR2 signaling in the bone metastasis of breast cancer cells (Sharma et al., 2019). The results of these previous studies might support the consequences of elevated expression levels of ELR^+^ CXC chemokines/receptors in bone metastasis of PC.

The results of the present study also demonstrated significantly higher expression levels of ELR^−^ CXC chemokines/receptors (CXCL9, CXCL13, CXCR3, and CXCR4) involved in lymphocyte chemotaxis in tumor cells from lymph node metastasis sites as well as in the primary lesions of patients with lymph node metastases (CXCL9, CXCL10, CXCL11, CXCR3, CXCR5, and CXCR7). Furthermore, based on the results of the comparative analysis of CXC chemokine/receptor gene expression patterns, a predictive model was developed, using both these factors and the clinical information currently used for risk assessment of patients with PC. The usefulness of this model as a predictor of lymph node metastases was evaluated. The results revealed that despite a slight increase in the AUC value, CXC chemokine/receptor expression was not a sufficient predictive biomarker of lymph node metastasis. However, the identification of CXCL10 as an independent predictor of regional lymph node metastasis and the association of CXCL10/CXCR3 co-expression with postoperative recurrence, suggests involvement of the ELR^−^ CXC chemokine/receptor axis in the mechanism of PC metastasis. Although ELR^−^ CXC chemokines belong to the antiangiogenic CXC chemokine family, ELR^−^ CXC chemokines, including CXCR3, CXCR4, CXCR5, CXCR6, and CXCR7, are associated with tumor growth, proliferation, and metastasis in PC (Keeley et al., 2010; Salazar et al., 2013). Particularly, previous studies have shown that CXCR3/CXCL10 signaling promotes metastasis in several cancer types (Wightman et al., 2015). CXCL10 induces chemotaxis of monocytes, natural killer cells, T lymphocytes, and various other subtypes of leukocytes through interactions with CXCR3, which is a G-protein-coupled seven-transmembrane receptor (Loetscher et al., 1998; Billottet et al., 2013). Several studies have reported that tumor cells expressing CXCR3 have increased ligand signaling before the onset of metastasis, which enhances the ability to metastasize (Kawada et al., 2007; Cambien et al., 2009; Ma et al., 2009; Wightman et al., 2015). Wightman et al. reported that the CXCL10/CXCR3 axis might be responsible for the metastasis of melanoma cells to the lungs and CXCL10/CXCR3 co-expression in melanoma as well as colon and renal cancers are associated with increased metastatic competence (Wightman et al., 2015). The results of the present study are consistent with those of the previous studies. Further, proinflammatory chemokines, including CXC chemokines, are transported via the lymphatic system to the draining lymph nodes (Palframan et al., 2001). Particularly, CXCL9 and CXCL10 expression in the lymph nodes is reported to promote CXCR3-mediated metastasis of melanoma cells (Kawada et al., 2004). The results of this and previous studies suggest that tumor cells expressing ELR^−^ CXC chemokines/receptors may be associated with the metastasis of PC and may use the normal pathways of lymphocyte chemotaxis to migrate to and invade lymph nodes.

The involvement of CXC chemokines in tumor metastasis was the primary focus of this research. However, chemokine-mediated signaling pathways, other than those involving CXC chemokines, are also suspected to be involved in tumor metastasis (Sarvaiya et al., 2013). The leading edge genes revealed by GSEA included chemokines other than CXC chemokines/receptors as well as various CC chemokines/receptors (CCR1, CCR3, CCR9, CCL1, CCL7, CCL15, CCL16, CCL23, CCL25, and CCL27), C chemokines/receptors (XCL1 and XCL2), and a CX_3_C chemokine/receptor (CX_3_CR1). Genes with ligand–receptor relationships included CCL7/CCR1, CCL7/CCR3, CCL15/CCR1, CCL15/CCR3, CCL16/CCR1, CCL23/CCR1, and CCL25/CCR9 (Supplementary Table 2). Notably, the expression levels of these chemokines/receptors were higher in bone metastasis than lymph node metastasis. Previous studies have reported that CCL25/CCR9 is associated with tumor migration, invasion, and antiapoptosis in PC (Singh et al., 2004; Sharma et al., 2010). Fractalkine/CX_3_CR1 has been associated with bone metastasis in PC (Jamieson et al., 2008). Further analysis of these chemokines would be helpful to reveal organ-specific genes involved in the metastasis of PC.

There were several limitations of this study that should be addressed. First, the cohorts of the cited studies were somewhat limited. Therefore, these findings should be further verified in other larger cohorts. In future work, we plan to determine whether the upregulation of ELR^+^ CXC chemokine/receptors in the primary tumors of patients with bone metastases is related to the development of bone metastases. Second, the data used in this study did not include information about splicing variants of CXCR. However, the expression of CXCR3A, a splicing variant of CXCR3, has been reported to promote PC, whereas CXCR3B might be a tumor suppressor (Wu et al., 2012). Further studies should consider the roles of splicing variants of CXCR3. Third, CXCL10 is an independent predictor of lymph node metastasis of the primary tumor, and co-expression of CXCL10/CXCR3 was associated with postoperative recurrence. However, in the present study, CXCL10 expression was not significantly higher in lymph node metastases than bone metastases (*p* = 0.1850). A possible reason for this discrepancy is that the metastases samples used in this study were collected from patients after receiving androgen deprivation therapy. A previous study reported that CXCL10 expression increased in response to androgens in rats (Asirvatham et al., 2006). Further studies should also consider interactions between CXC chemokines/receptors and androgen deprivation therapy.

## Conclusions

The results of the present study demonstrate that CXC chemokine/receptor gene expression patterns in patients with PC are heterogeneous and differ according to the metastatic site, suggesting that CXC chemokines/receptors may contribute to the mechanism of organ-specific metastasis of PC. Further, these findings will require validation in separate cohorts as well as verification using both cell and animal models.

## Data Availability Statement

Publicly available datasets were analyzed in this study. This data can be found here: cBioPortal for Cancer Genomics (https://www.cbioportal.org/).

## Ethics Statement

Ethical review and approval was not required for the study on human participants in accordance with the local legislation and institutional requirements. Written informed consent for participation was not required for this study in accordance with the national legislation and the institutional requirements.

## Author Contributions

NN designed analytical methods, analyzed the data, and wrote the manuscript. GL, SH, and IK revised the manuscript. All authors read and approved the final manuscript. All authors contributed to the article and approved the submitted version.

## Conflict of Interest

The authors declare that the research was conducted in the absence of any commercial or financial relationships that could be construed as a potential conflict of interest.

## Supplementary Material

Supplementary Figure 1 A flowchart of the data retrieval process, indicating the type of analysis for specific datasets.

Supplementary Figure 2 Lists of the tissue collection sites, and the number of samples collected from each site. Comparative analysis of serum PSA levels and radical prostatectomy Gleason scores of patients with bone vs. lymph node metastases.

Supplementary Figure 3 CXC chemokine/receptor gene expression levels at all metastatic sites. The box plots indicate mRNA expression levels of individual samples.

Supplementary Table 1 Gene set enrichment analysis of differences in KEGG pathway enrichment between bone metastases and lymph node metastases of PC. This table shows the result of gene set enrichment analysis (GSEA), including SIZE, enrichment score (ES), normalized enrichment score (NES), normalized *p*-value (NOM *p*-val), false discovery rate *q*-value (FDR *q*-val), family-wise error rate *p*-value results (FWER *p*-val), Rank at max. SIZE represents the number of genes in the gene set after filtering out those genes not in the expression dataset. ES is the degree to which this gene set is overrepresented at the top or bottom of the ranked list of genes in the expression dataset. NES is the enrichment score for the gene set after it has been normalized across analyzed gene sets. NOM *p*-val is the statistical significance of the enrichment score. FDR *q*-val is the estimated probability that the normalized enrichment score represents a false positive finding. FWER *p*-val is a more conservatively estimated probability that the normalized enrichment score represents a false positive finding. Rank at max represents the position in the ranked list at which the maximum enrichment score occurred. In the Olfactory transduction pathway, Neuroactive ligand receptor interaction pathway, Extracellular matrix (ECM) receptor interaction pathway, Cytokine–Cytokine receptor interaction pathway, Drug metabolism cytochrome p450 pathway, Hematopoietic cell lineage pathway, Retinol metabolism pathway, Taste transduction pathway, Autoimmune thyroid disease pathway, and Metabolism of xenobiotics by cytochrome P450 pathway, the *p*-value is significantly small (*p* < 0.01).

Supplementary Table 2 Gene set enrichment analysis of the distribution of the expression of the 262 KEGG Cytokine–Cytokine Receptor Interaction pathway component genes. This table includes Gene symbol, Rank in gene list, Rank metric score, Running enrichment score (ES), and Core enrichment. Ranking in gene list represents position of the gene in the ranked list of genes. Rank metric score represents score used to position the gene in the ranked list. Running ES represents the enrichment score at this point in the ranked list of genes. Genes with a Yes value in core enrichment column contribute to the leading-edge subset within the gene set. The 99 leading edge genes were included in Cytokine-Cytokine Receptor Interaction pathway component genes. Among them, five gene are CXC chemokines/ receptors (CXCL1, CXCL3, CXCL6, CXCR1, and CXCR2).
