# Supplementary figures and images for: CXC Chemokine/Receptor Axis Profile and Metastasis in Prostate Cancer
Source: Front Mol Biosci. 2020 Oct 15;7:579874. doi: 10.3389/fmolb.2020.579874 (PMC7593595; doi:10.3389/fmolb.2020.579874)

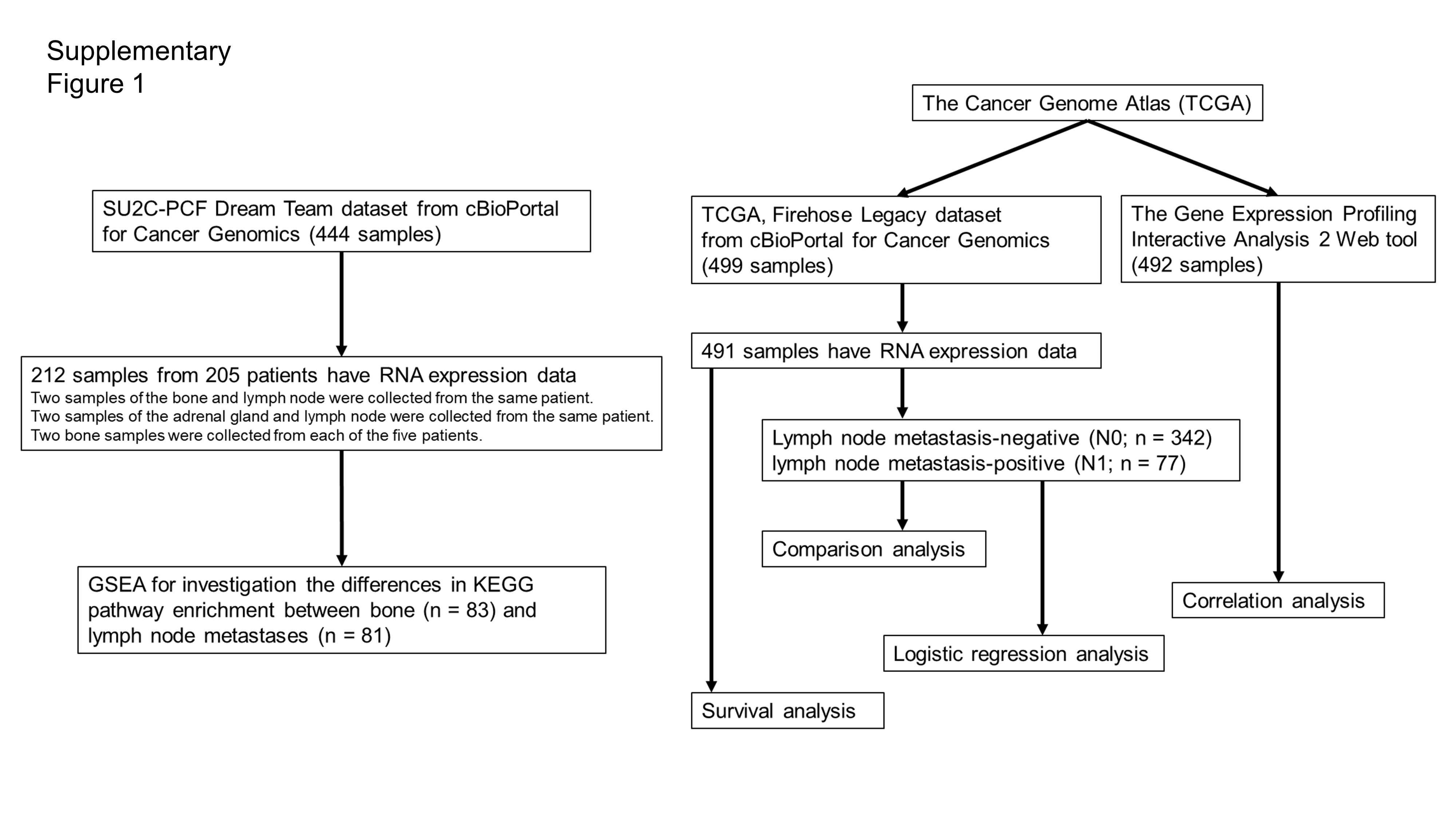

Supplement: Supplementary Figure 1 — A flowchart of the data retrieval process, indicating the type of analysis for specific datasets. [file Image_1.TIF]

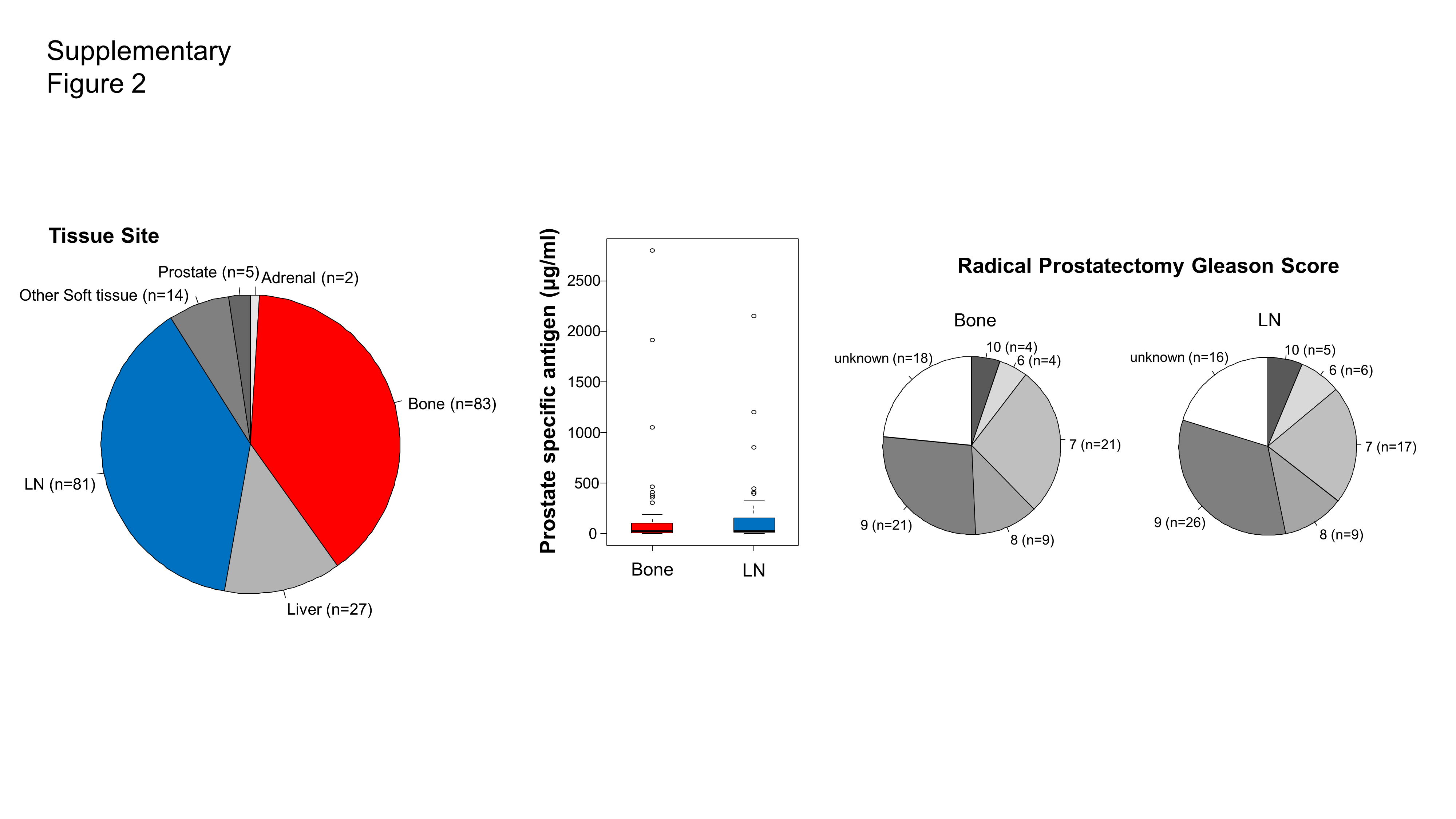

Supplement: Supplementary Figure 2 — Lists of the tissue collection sites, and the number of samples collected from each site. Comparative analysis of serum PSA levels and radical prostatectomy Gleason scores of patients with bone vs. lymph node metastases. [file Image_2.TIF]

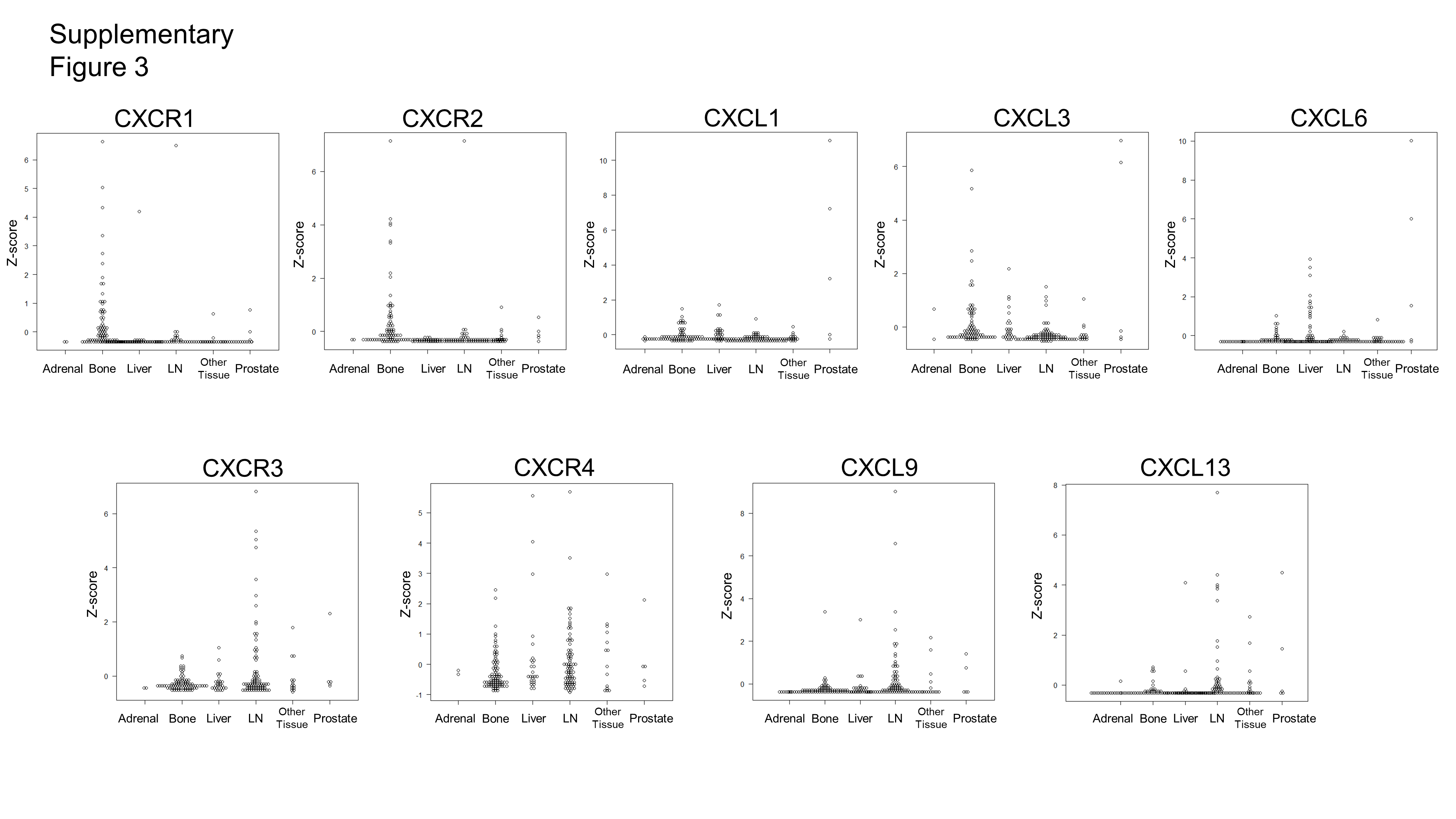

Supplement: Supplementary Figure 3 — CXC chemokine/receptor gene expression levels at all metastatic sites. The box plots indicate mRNA expression levels of individual samples. [file Image_3.TIF]
